# Tm-doped TiO_2_ and Tm_2_Ti_2_O_7_ pyrochlore nanoparticles: enhancing the photocatalytic activity of rutile with a pyrochlore phase

**DOI:** 10.3762/bjnano.6.62

**Published:** 2015-03-02

**Authors:** Desiré M De los Santos, Javier Navas, Teresa Aguilar, Antonio Sánchez-Coronilla, Concha Fernández-Lorenzo, Rodrigo Alcántara, Jose Carlos Piñero, Ginesa Blanco, Joaquín Martín-Calleja

**Affiliations:** 1 Departamento de Química Física, Facultad de Ciencias, Universidad de Cádiz, E-11510 Puerto Real (Cádiz), Spain; 2 Departamento de Ciencias de los Materiales, Ingeniería Metalúrgica y Química Inorgánica, Facultad de Ciencias, Universidad de Cádiz, E-11510 Puerto Real (Cádiz), Spain

**Keywords:** nanoparticles, photocatalysis, pyrochlore, titanium dioxide, thulium

## Abstract

Tm-doped TiO_2_ nanoparticles were synthesized using a water-controlled hydrolysis reaction. Analysis was performed in order to determine the influence of the dopant concentration and annealing temperature on the phase, crystallinity, and electronic and optical properties of the resulting material. Various characterization techniques were utilized such as X-ray diffraction, Raman spectroscopy, X-ray photoelectron spectroscopy and UV–vis spectroscopy. For the samples annealed at 773 and 973 K, anatase phase TiO_2_ was obtained, predominantly internally doped with Tm^3+^. ICP–AES showed that a doping concentration of up to 5.8 atom % was obtained without reducing the crystallinity of the samples. The presence of Tm^3+^ was confirmed by X-ray photoelectron spectroscopy and UV–vis spectroscopy: the incorporation of Tm^3+^ was confirmed by the generation of new absorption bands that could be assigned to Tm^3+^ transitions. Furthermore, when the samples were annealed at 1173 K, a pyrochlore phase (Tm_2_Ti_2_O_7_) mixed with TiO_2_ was obtained with a predominant rutile phase. The photodegradation of methylene blue showed that this pyrochlore phase enhanced the photocatalytic activity of the rutile phase.

## Introduction

TiO_2_ is one of the most efficient semiconductors used as a photocatalyst for the degradation of organic compounds. This is due to its high chemical and biological stability, low cost, excellent electronic and optical properties, and the strong oxidation capacity of its photogenerated holes [1–2]. Photocatalytic activity depends on several catalytic properties, such as band gap energy, specific surface area, the extent of crystallinity, the structure of the material, etc. [3]. In general, a good photocatalyst should efficiently absorb photons with an energy equal to or higher than its band gap, thus generating an electron–hole pair. These electrons and holes react with oxygen, water or hydroxy groups to produce highly reducing or oxidizing species that are capable of degrading different organic and inorganic compounds [4]. For this reason, there have been many studies on the synthesis of TiO_2_ regarding the control of its physical and chemistry properties. For example, the TiO_2_ doping and the annealing temperatures have been widely studied to control its crystalline structure, optical and electronic properties, etc. Marschall and Wang reported doping with non-metals such as boron, carbon, nitrogen, fluorine, iodine, phosphorus, or sulfur [5]. TiO_2_ doping with metallic elements has also been reported, using for example niobium [6], silver [7] or copper [8] as dopants. Many pyrochlore-type compounds (A_2_B_2_O_7_) have been studied to evaluate their semiconductor properties for photocatalytic applications. For example, it is possible to find studies in the literature in which pyrochlore-type compounds with high photocatalytic activity are used as a photocatalysts, such as Bi_2_Ti_2_O_7_ [9–10], Pb_2_Nb_2_O_7_ [11]; other pyrochlore compounds that have been evaluated are rare earths, such as Gd_2_BiSbO_7_ [12] or Ln_2_Ti_2_O_7_ (Ln = Nd, Gd, Er) [13].

In this study, Tm-doped TiO_2_ nanoparticles were synthesized using a water-controlled hydrolysis reaction. The effect of the dopant concentration and the annealing temperature on the resulting phase, crystallinity, and electronic and optical properties was analyzed. A pyrochlore phase (Tm_2_Ti_2_O_7_) was observed in the samples annealed at 1173 K. In turn, the influence of this pyrochlore phase on the photocatalytic activity of rutile-phase TiO_2_ was analyzed by a study of the photodegradation of methylene blue.

## Experimental

The synthesis method was based on the hydrolysis reaction of titanium(IV) isopropoxide, where the amount of reaction water added was controlled. The method is summarized as follows. A stoichiometric amount of thulium chloride (purity 99.9%, Sigma-Aldrich) was dissolved in water (7 mL) to obtain a theoretical Tm/TiO_2_ doping of 5, 10 and 15 wt %. Then, this solution was added dropwise under sonication (130 W, 20 Hz) to titanium(IV) isopropoxide (10 mL, purity 97%, Sigma-Aldrich) for 5 min. To synthesize pure TiO_2_ nanoparticles, only water (7 mL) was added. The reaction conditions were 323 K and pH 4. The solvent was evaporated by means of a progressive increase in the oven temperature until the annealing temperature was reached. These temperatures were 773, 973 and 1173 K, for the three samples, respectively. This was followed by annealing in air for 2 h.

The samples were characterized to quantify their composition, structure and optical properties. Pure TiO_2_ samples were synthesized and characterized to study the effect of Tm in the bulk TiO_2_. The instrumental techniques and the procedures used are described below.

The amount of Tm in the doped samples was determined by inductively coupled plasma atomic emission spectroscopy (ICP–AES, Iris Intrepid spectrometer, Thermo Elemental). The procedure for the analysis was based on the acid extraction of the samples and was performed in duplicate. Moreover, X-ray diffraction (XRD) was used to determine the crystalline phases in the samples. The tests were performed using a diffractometer (Bruker, model D8 Discover), with Cu Kα radiation. The 2θ scan conditions were from 20 to 75° with a resolution of 0.025°, taken at 40 kV and 30 mA. From the patterns obtained, the anatase and rutile mass fraction, the average crystallite size, the unit cell volume and the specific surface area were semiquantitatively estimated. The mass fraction of the anatase phase was calculated using the following equation:

[1]
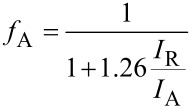


where *I*_A_ is the intensity of the reflection of the (101) plane for the anatase phase, and *I*_R_ is the intensity of the reflection of the (110) plane for the rutile phase [14]. To determine the mass fraction of the rutile phase (*f*_R_), only the presence of the anatase and rutile phases was considered. Additionally, the average crystallite size (*t*) was obtained using the Scherrer equation,

[2]
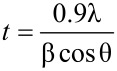


where λ is the wavelength of X-ray radiation (Cu Kα, 0.154 nm), β is the full width at half maximum (FWHM) of the peak, and θ is the diffraction angle [15]. The values of θ and β used are those corresponding to the major peaks of each phase, that is, planes (101) and (110) for anatase and rutile phases, respectively. A baseline correction was performed to obtain the best value of *t*. The volume of the unit cell (*V*) was calculated and used to obtain the specific surface area (*s*_a_). The values of *V* were obtained from the values of the lattice constant as *V* = *ac*^2^ (*a* = *b* ≠ *c*), for the anatase and rutile phases [16]. The values of *a* and *c* were estimated from the two peaks with the highest intensity for each phase, and using the equation typical for tetragonal systems [17],

[3]
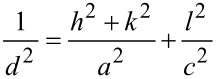


where *d* is the interplanar distance, and *h*, *k*, *l* are the Miller indices of the planes used. Finally, *s*_a_ was calculated using the equation [16] *s*_a_ = 6/(*t*ρ), where ρ is the density obtained by XRD as [16] ρ = *nM*/(*NV*), where *N* is Avogadro’s number, *M* is the molecular weight, *n* = 2 for the rutile phase, *n* = 4 for the anatase phase, and *V* is the unit cell volume calculated as described above. The molecular weight was calculated considering the empirical formula obtained from ICP–AES and X-ray photoelectron spectroscopy (XPS) results. XPS was used to analyze the chemical composition and the chemical bonding states of the samples. A spectrometer (Kratos Axis UltraDLD) with monochromatic Al Kα radiation (1486.6 eV) and a 20 eV pass energy was used to record the XPS spectra. The binding energy scale was referenced to the C1s signal at 284.8 eV and given with an accuracy of 0.1 eV. Moreover, to study the optical properties of the samples and to determine the band gap energy, UV–vis spectroscopy in diffuse reflectance (DR–UV–vis) mode was used. The custom-built system was composed of: (i) an integrating sphere (Spectra Tech), (ii) a spectrometer (USB2000+, Ocean Optics), and (iii) a xenon lamp (Spectral Products, ASB-XE-175) as an illumination source. The Kubelka–Munk formalism and Tauc plots were used to determine the band gap energy. The Kubelka–Munk function for diffuse reflectance (*R*) is

[4]
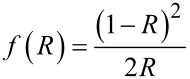


For a semiconductor, the plot of [*f*(*R*)·*h*ν]*^n^* versus *h*ν shows a linear region for *n* = ½ if the band gap is determined by direct transition, or for *n* = 2 if the transition is indirect [18–19]. As the TiO_2_ band gap is determined by direct transition, the plot of [*f*(*R*)·*h*ν]^1/2^ versus *h*ν shows a linear region which satisfies the equation

[5]
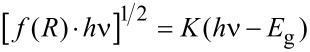


where *h*ν is the photon energy, *E*_g_ is the band gap energy, and *K* a characteristic constant for the semiconductor. Raman spectroscopy was used to study the vibrational properties of the samples synthesized. Raman spectra were recorded in a backscattering geometry using a double monochromator (Jobin Yvon, U10009) equipped with a photomultiplier tube (Hamamatsu, R-943), and a diode-pumped, solid state, 532 nm laser (CNI, MSL-III-532nm-50mW). The Raman study was carried out using a portable system (B&W Tek Raman, *i*-Raman) equipped with a diode laser (BAC100-785C, 785 nm) as an illumination source. Additionally, transmission electron microscopy (TEM) imaging and electron energy loss spectroscopy (EELS) analysis were carried out on samples of different Tm concentrations (2 and 5.8 atom %) which underwent different annealing conditions in order to evaluate the Tm distribution in the nanoparticle. As EELS provides information relating the material with the resulting electron inelastic scattering, this technique is sensitive to the atomic composition, valence and conduction band electronic properties, surface properties and chemical bonding. The latter allows for the characterization of the structural properties of the lattice traversed by the electron beam and, thus, determination of the crystalline phases. Moreover, the sensitivity to atomic composition allows for differentiation of the location of the particle where Tm is located. For this analysis, EELS data were collected in scans by crossing different particles and EELS maps of Ti and Tm were acquired and compared in order to identify the relative proportion and location of Tm in the nanoparticle. TEM and EELS data were obtained using a beam of an electron microscope (JEOL-2010F) with a nominal probe size of 1 nm.

Finally, the photocatalytic activity of the pure and Tm-doped TiO_2_ nanoparticles was analyzed. A study of the photodegradation of methylene blue (MB) was performed using a set of five actinic lamps emitting at around 360 nm. The initial concentration of the aqueous solution of MB (purity 82%, Panreac) was 1.56 · 10^−5^ M, and the concentration of the photocatalyst was 0.3 g·L^−1^. The photocatalyst/MB mixture was kept in darkness for 30 min, and the reaction time was 2 h. The photodegradation of MB was studied by absorbance measurements. A calibration curve was used to determine the evolution of the MB concentration and the kinetics of the photodegradation. The absorbance was measured by using a spectrometer (Ocean Optics, USB4000+) with a UV–vis–NIR light source (Ocean Optics, DH-2000-BAL).

## Results and Discussion

### Inductively coupled plasma–atomic emission spectroscopy

Table 1 shows the weight percentage values of Tm with respect to TiO_2_, obtained by ICP–AES and calculated as the arithmetic average of the values obtained for different annealing temperatures. Additionally, the atomic percentage and the atomic/molar ratio of Tm/(Tm+Ti) were calculated, resulting in values of 2.0, 4.5 and 5.8 atom % for the theoretical doping masses of 5, 10 and 15 wt %, respectively. The resulting doping concentrations were not as high as those reported for other ions (of up to 10 atom %) because the Tm^3+^ ion is substantially larger than the Ti^4+^ ion that it replaces in the crystalline lattice, as will be discussed below. The atomic percentage values are used in the discussion below to determine the dependence of the material properties on the doping concentration.

**Table 1:** Experimental weight percentage obtained by ICP–AES and atomic/molar percentage compared to the theoretical, stoichiometric weight percentage.

Theoretical wt % (Tm/TiO_2_)	Measured wt % (Tm/TiO_2_)	Atom % (Tm/(Tm + Ti))

Pure TiO_2_	0.0	0.0
5.0	4.36 ± 0.07	2.0
10.0	9.90 ± 0.10	4.5
15.0	12.90 ± 0.15	5.8

### X-ray diffraction

**Figure 1:**
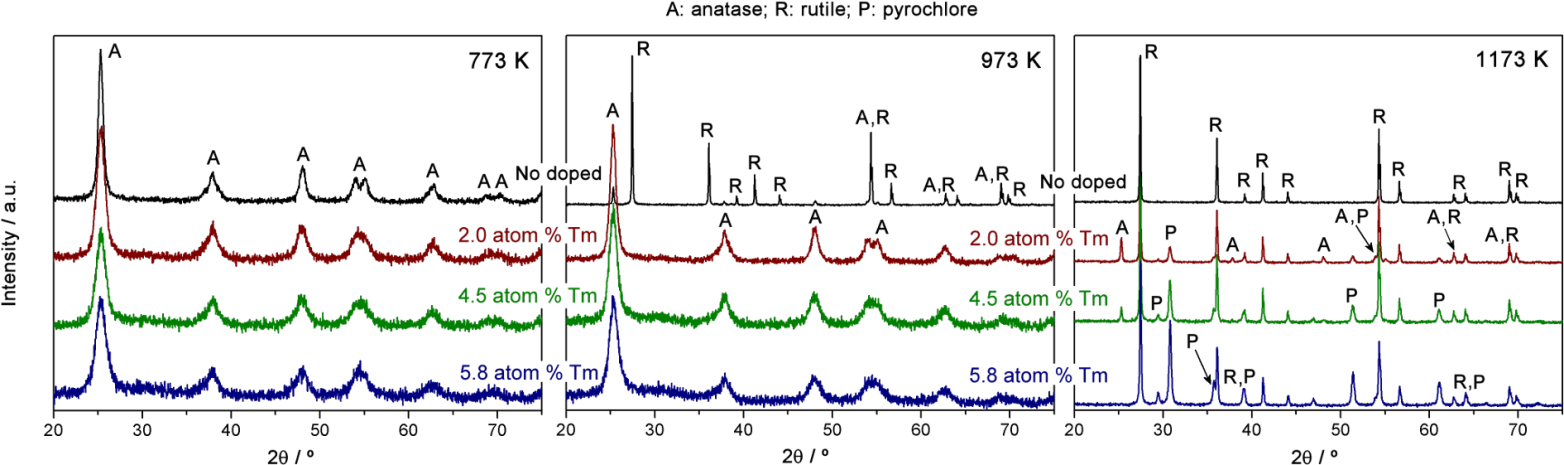
XRD patterns of the samples synthesized.

Figure 1 shows the XRD patterns of the synthesized samples. In accordance with the references, JCPDS 21-1272 for anatase and JCPDS 21-1276 for rutile, the peaks of both phases of TiO_2_ are identified in Figure 1. The samples annealed at 773 K were exclusively composed of the anatase phase while the sample of pure TiO_2_ annealed at 973 K consisted of mainly a rutile phase, but also contained some anatase phase. At 973 K the effect of Tm doping was confirmed by the presence of the predominance of the anatase phase. Finally, at 1173 K, peaks corresponding to the rutile phase, residual anatase phase, and a new crystalline phase were observed, as indicated by “P” in Figure 1. In accordance with the JCPDS 23-0590 reference spectra, the new peaks were assigned to a pyrochlore oxide, Tm_2_Ti_2_O_7_ [20]. Pyrochlore is a superstructure of the ideal defect fluorite structure A_4_X_7_ with 1/8 anion deficiency [21]. The presence of other Tm species in the XRD patterns was tested, such as the fluorite phase of Tm_2_Ti_2_O_7_ or any phase of Tm_2_O_3_. The main peak of the fluorite phase is commonly observed at 2θ = 30° [22], while for Tm_2_O_3_ it appears at 2θ = 29.46° [23]. Therefore, the XRD patterns do not show evidence of fluorite or any crystalline phase of Tm_2_O_3_. Thus, we can conclude that in the Tm-doped samples annealed at 773 K and 973 K, the predominant phase obtained is anatase, internally doped with Tm^3+^. The doped samples annealed at 1173 K possibly contain undoped rutile and pyrochlore phases. Shlyakhtina et al. reported fluorite–pyrochlore–fluorite phase transitions for RE_2_Ti_2_O_7_ (where RE = Lu, Yb, Tm), and that a pyrochlore structure is formed at T > 1073 K [24], which is consistent with the results obtained for our samples. The presence of this pyrochlore phase is of great interest in photocatalytic applications, as will be discuss below.

A semiquantitative analysis was performed using the patterns obtained. Table 2 shows the mass fraction values of the anatase and rutile phases, average crystallite size, unit cell volume and specific surface area for each phase in each sample, calculated following the procedure described in the Experimental section. Only the presence of anatase and rutile phases was considered to study the mass fraction. From the calculated values of *f*_A_, it is observed that the anatase and rutile phases do not coexist in the samples. Moreover, for both phases, the higher the annealing temperature, the higher the *t*, due to the sintering of the nanocrystals. Generally, *t* decreases with an increased dopant concentration. The introduction of a Tm^3+^ ion into the structure can create structural distortions that break the crystal continuity [8,24]. This occurs in all cases except for the anatase phase at an annealing temperature of 1173 K, in which an increase in the average crystallite size is observed. Finally, the *s*_a_ trend in relation to the annealing temperature and the Tm-dopant concentration is the inverse of the trend shown for *t*, as is well-known.

**Table 2:** Semiquantitative values of anatase and rutile mass fraction, average crystallite size, unit cell volume and specific surface area obtained from XRD patterns of the samples synthesized.

Atom % Tm/(Tm + Ti)	*T*_a_/K	*f*_A_/wt %	*f*_R_/wt %		*t*/nm	*V*/Å^3^	*s*_a_/m^2^·g^−1^

0.0	773	100.0	0.0	anatase	13.7	136.6	113.0
	973	8.4	91.6	anatase	59.0	135.9	26.0
				rutile	66.3	62.3	21.3
	1173^a^	0.0	100.0	rutile	69.9	62.4	20.2

2.0	773	100.0	0.0	anatase	8.2	131.1	175.7
	973	100.0	0.0	anatase	11.9	138.5	127.5
	1173^a^	12.8	87.2	anatase	41.4	135.3	35.9
				rutile	59.6	62.5	23.0

4.5	773	100.0	0.0	anatase	7.3	131.6	189.6
	973	100.0	0.0	anatase	8.6	134.2	165.8
	1173^a^	6.1	93.9	anatase	54.9	136.0	26.2
				rutile	55.9	62.4	23.6

5.8	773	100.0	0.0	anatase	6.5	140.3	222.7
	973	100.0	0.0	anatase	7.9	137.5	179.3
	1173^a^	0.0	100.0	rutile	52.1	62.2	24.6

^a^At 1173 K, only the presence of anatase and rutile phases is considered to estimate the values of *f*_A_ and *f*_R_.

### X-ray photoelectron spectroscopy

**Figure 2:**
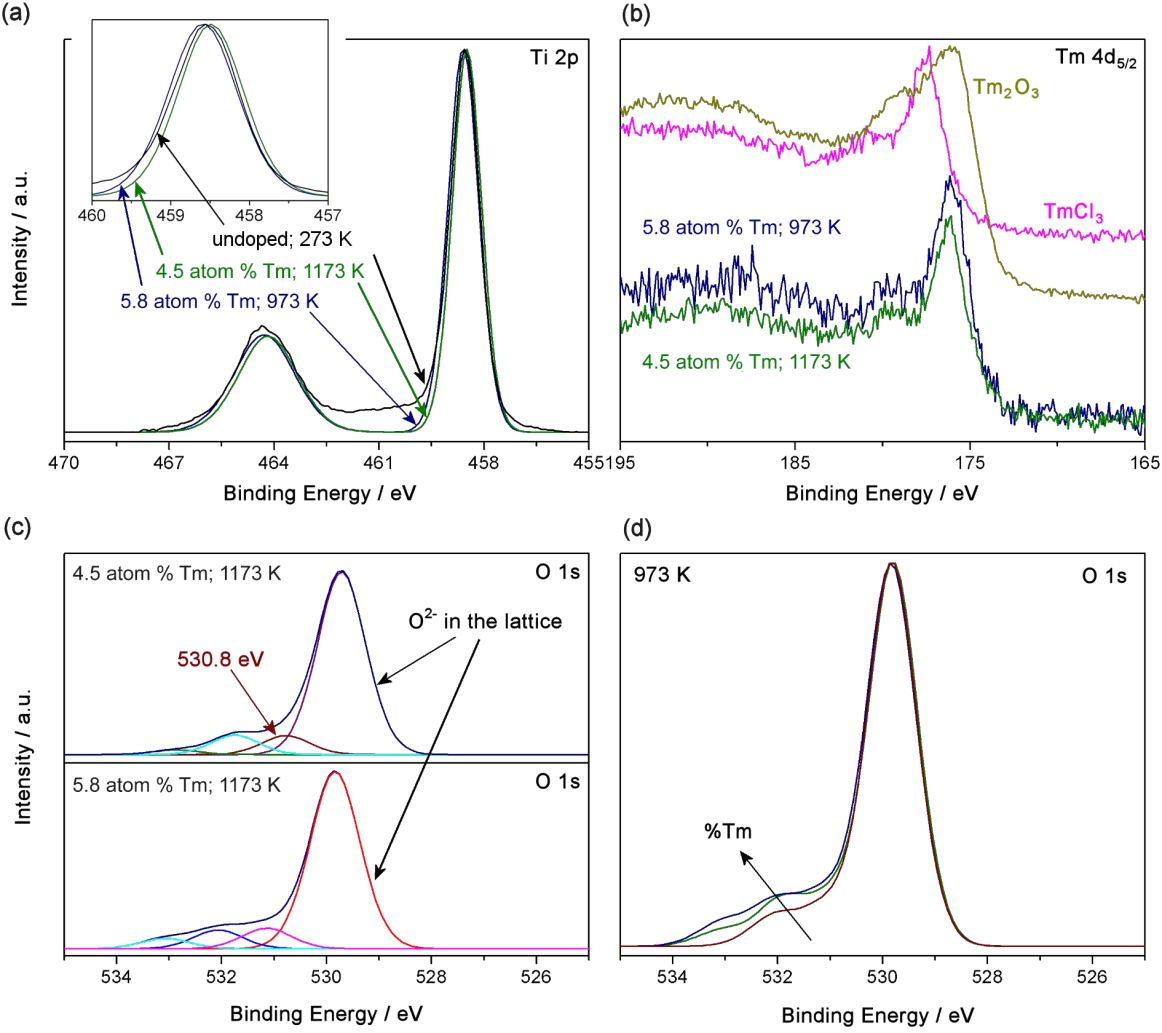
XPS spectra of Ti 2p (A); Tm 4d_5/2_ (B); O 1s including contributions of different O in the samples (C) and O 1s versus percentage of Tm (D).

Figure 2a shows the Ti 2p spectra of three samples considered as representative: pure TiO_2_ annealed at 973 K, and 4.5 and 5.8 atom % Tm-doped TiO_2_ annealed at 1173 and 973 K, respectively. The binding energy (BE) for Ti 2p_3/2_ was near 458.5–458.7 eV. The typical values reported for Ti(IV) and Ti(III) are 458.66 and 457.13 eV, respectively [25]. Thus, the values obtained here are consistent with those reported for Ti(IV). Also, the energy difference between the BE of Ti 2p_3/2_ and Ti 2p_1/2_ was around 5.7–5.8 eV, which is also consistent with values reported in the literature for Ti(IV) [26]. The FWHM of the Ti 2p_3/2_ peak (0.98 eV) is quite consistent with this assignment [27]. Therefore, the predominant oxidation state for Ti in our samples was assumed to be Ti^4+^, while the presence of Ti^3+^ in TiO_2_ [28] was negligible.

The BE for Tm 4d_5/2_ in the doped samples was around 176.5 eV (see Figure 2b), which is consistent with the value for Tm_2_O_3_ reported by Uwamino et al. [29]. Moreover, two reference samples (TmCl_3_ and Tm_2_O_3_) were used to compare the doped samples (see Figure 2b). The BE for Tm 4d_5/2_ for TmCl_3_ and Tm_2_O_3_ was 177.5 and 176.3 eV, respectively, confirming the presence of Tm(III) in an oxide matrix.

Thus, considering that the predominant cations in the TiO_2_ lattice of the doped samples are Ti^4+^ and Tm^3+^, and that the doping is substitutional, oxygen vacancies are generated to maintain the local neutrality of the lattice. The presence of oxygen vacancies is interesting for photocatalytic applications because, for example, an increase in oxygen vacancies generates more surface defects on the synthesized nanoparticles. This can be studied from the O 1s XPS spectra. Figure 2c (bottom) shows the different contributions in the O 1s spectrum for the 5.8 atom % Tm-doped TiO_2_ sample annealed at 973 K, which is considered as representative of the spectra obtained. The most intense contribution appeared around 529.8 eV and is assigned to O^2−^ in the TiO_2_ lattice. The other contributions are assigned to adsorbed species, such as hydroxy groups, or water [30–31]. The top part of Figure 2c shows the spectrum of O 1s for the 4.5 atom % Tm-doped TiO_2_ sample annealed at 973 K in which a new contribution to the O 1s signal was observed at a BE of 530.8 eV. This signal is assigned to O^2−^ in the Tm_2_Ti_2_O_7_ pyrochlore lattice [32]. Figure 2d demonstrates how the amount of O in the adsorbed species increases with the Tm concentration. This is due to the increase in oxygen vacancies produced, and thus in the number of adsorption centers available. The adsorption of hydroxy groups onto the surface is of interest in photocatalytic applications. Surface hydroxy groups can react with photogenerated holes, inhibiting the recombination of an electron–hole pair, and thus enhancing charge transfer [33]. Additionally, they can also lead to the production of hydroxyl radicals, a highly oxidizing species in the degradation of organic compounds [34].

### UV–vis spectroscopy

**Figure 3:**
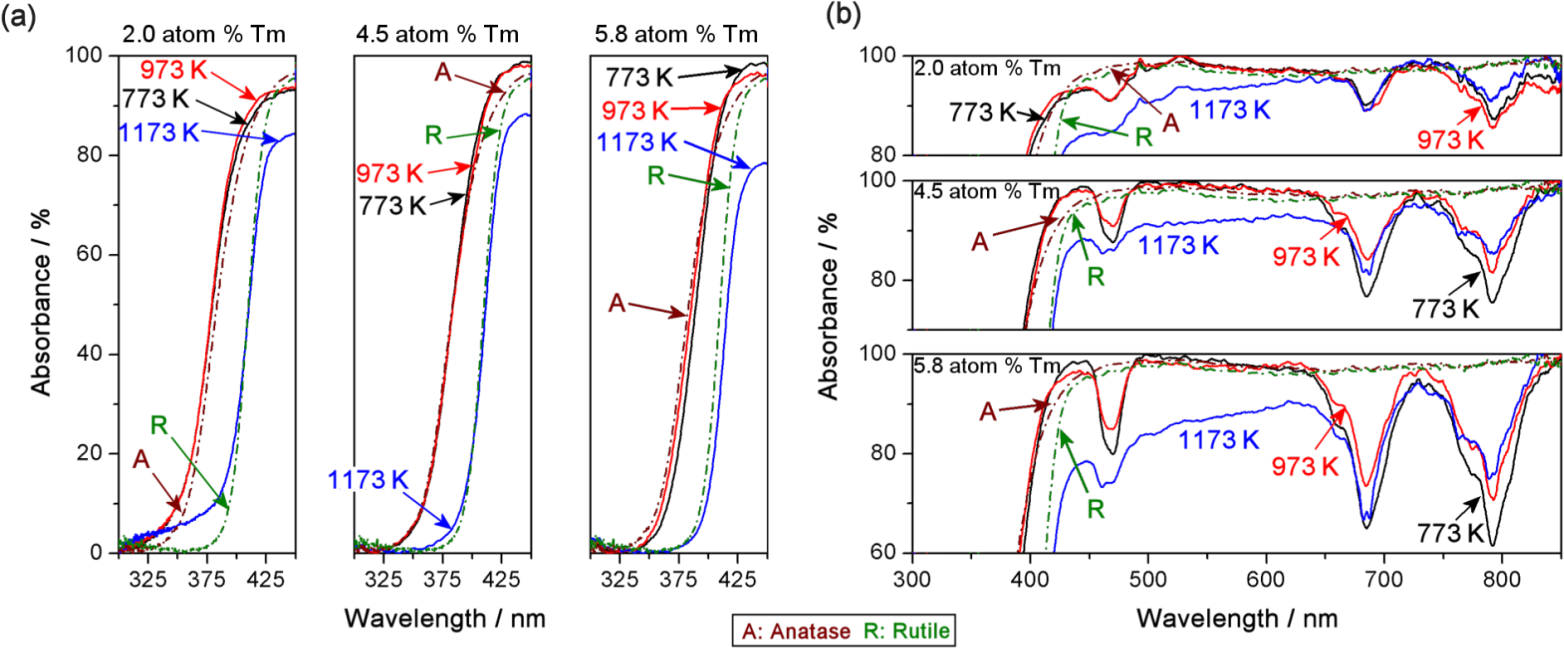
UV–vis spectra of the synthesized samples obtained in diffuse reflectance mode.

A redshift of the main absorption band assigned to TiO_2_ is observed in the samples annealed at 1173 K (see Figure 3a). This is due to the predominant rutile crystalline phase in these samples, which has a lower band gap energy than anatase [35]. Moreover, three absorption bands (Figure 3b) are clearly seen centered about 468 nm, 685 nm and 792 nm for all the samples, where the intensity increases with higher Tm doping. The three bands correspond to the transitions of ^3^H_6_(Tm^3+^)→^1^G_4_(Tm^3+^), ^3^H_6_(Tm^3+^)→^3^F_3_(Tm^3+^) and ^3^H_6_(Tm^3+^)→^3^H_4_(Tm^3+^), respectively [36].

**Figure 4:**
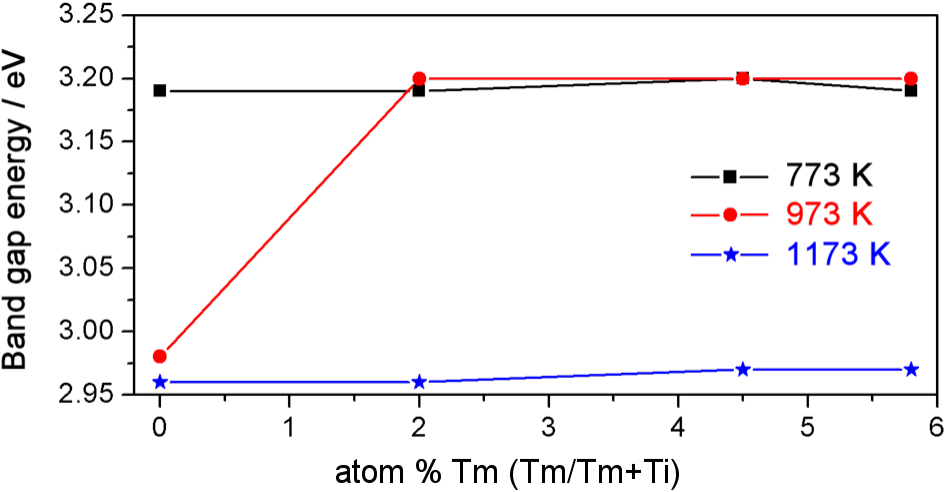
Band gap energy values as a function of Tm doping concentration at different annealing temperatures.

The band gap energy for the main transition in the semiconductor was determined as described in the Experimental section. Figure 4 shows the evolution of the band gap energy values as a function of doping concentration and annealing temperature. Lower values were obtained (≈0.2 eV) for the samples in which the predominant phase was rutile, which has a lower band gap than the anatase phase [35]. For the samples which have only one predominant phase, no changes were observed in the band gap energy dependence.

### Raman spectroscopy

**Figure 5:**
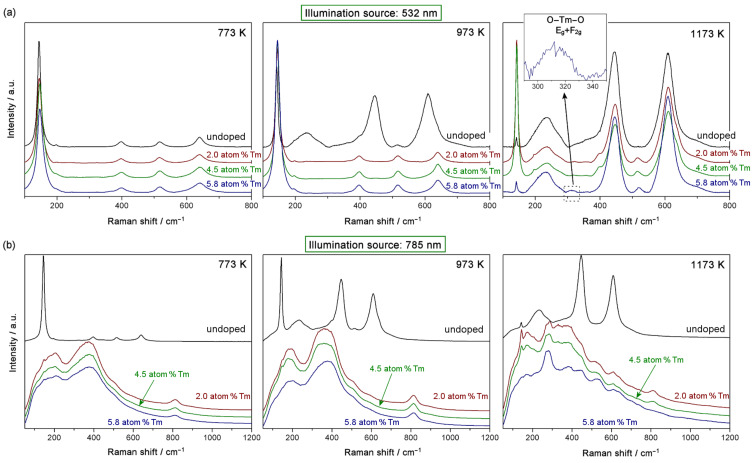
Raman spectra obtained using a 532 nm (a) and a 785 nm (b) illumination source.

The Raman spectra given in Figure 5 were recorded using two illumination sources, a green laser emitting at 532 nm and a red laser emitting at 785 nm. The anatase phase shows six Raman active modes: 144 cm^−1^ (E_g_), 197 cm^−1^ (E_g_), 399 cm^−1^ (B_1g_), 519 cm^−1^ (A_1g_+B_1g_) and 639 cm^−1^ (E_g_) [37]. The rutile phase shows four active modes at: 143 cm^−1^ (B_1g_), 447 cm^−1^ (E_g_), 612 cm^−1^ (A_1g_) and 826 cm^−1^ (B_2g_) [38]. From the spectra obtained with the green laser and the active modes noted above, the primary phase in the samples was qualitatively deduced. The anatase phase was predominant in the samples annealed at 773 K and in all of the doped samples annealed at 973 K. In contrast, the rutile phase was predominant in all of the samples annealed at 1173 K and in the undoped sample annealed at 973 K. We thus conclude that the results obtained from the Raman spectra are consistent with the XRD results.

The XRD results allowed for the confirmation of a pyrochlore phase in the samples annealed at 1173 K. The pyrochlore structure has six active Raman modes: Γ = A_1g_ + E_g_ + 4 F_2g_ [21,39]. The most intense band usually appears at approximately 304–308 cm^−1^ and is assigned to an O–RE–O bending mode, and consists of two modes with a similar frequency (E_g_, F_2g_) [39]. The relative intensity of the pyrochlore bands in relation to the bands of pure TiO_2_ is very low, and only a weak band is observed centered at ≈312 cm^−1^. This band is shown in the inset in Figure 5a for the 5.8 atom % Tm-doped sample annealed at 1173 K. As with XRD, the pyrochlore phase only appears at this temperature, and the intensity of the band increases with doping concentration.

Figure 5b shows the Raman spectra obtained using red laser illumination. These spectra are clearly different from those obtained with the green laser (see Figure 5a). The doped samples have an absorption band at ≈790 nm, as was demonstrated by DR–UV–vis measurements. This band is energetically very close to the excitation source used in Raman spectroscopy (785 nm). The luminescence phenomena associated with Tm^3+^are well-documented [20,23,36,40–41]. At this excitation wavelength, it is assumed that photon absorption occurs, which then results in the luminescence processes. The ^3^H_4_ excited state is accessible with this laser excitation (see UV–vis results), and electrons in this state can transition to the lower levels of the ^3^H_6_ ground state, generating luminescence. The shape of the resulting Raman signal is typical of luminescence processes: a very wide band (in contrast to the sharp, characteristic, Raman bands) is observed. When the electronic transition that generates the luminescence process takes place between two triplet states, absorption (and thus the relaxation in luminescence) processes are permitted, and intense signals are generated. This can be excited with the 785 nm illumination source where the permitted transitions are generated. Additionally, it is noted that the signal associated with luminescence is not observed in any of the pure TiO_2_ samples annealed at any of the three temperatures. Furthermore, for the samples annealed at 1173 K (where the Tm appears in pyrochlore form and there is only rutile phase), the luminescence signal is weaker and the spectrum peaks are more distinct with Raman signal peaks clearly evident. As reported in the literature [23,42], these luminescence processes may be of interest in several photocatalytic and photovoltaic applications as they can improve the photon absorption efficiency of semiconductors. Moreover, the luminescence phenomena are typically related to recombination processes, so the higher luminescence signal, the higher the recombination process. Thus, as the samples comprised of a pyrochlore phase show a weaker luminescence signal, the recombination processes in these samples are correspondingly lower. This is a very important concept with respect to the photocatalytic tests discussed below.

### TEM and EELS analysis

**Figure 6:**
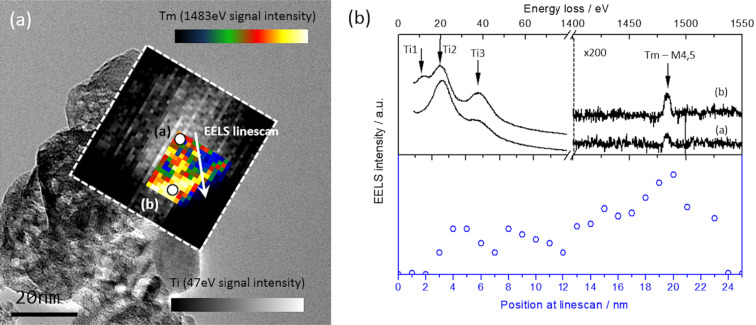
(a) TEM image of 2 atom % Tm-doped sample annealed at 1173 K and EELS maps obtained for the Ti3 peak (grey scale map) and Tm-M_4,5_ signal (temperature map). (b) EELS spectra obtained at positions (a) and (b) in Figure 6a, corresponding to the core and edge of the nanoparticle. Also given is the Tm-M_4,5_ signal intensity profile along the corresponding linescan shown in Figure 6a.

TEM–EELS analysis was performed using a 2.0 atom % Tm-doped TiO_2_ sample annealed at 1173 K and a 5.8 atom % Tm-doped TiO_2_ sample annealed at 973 K. Figure 6 summarizes the results obtained for the 2.0 atom % Tm-doped TiO_2_ sample annealed at 1173 K that corresponds to a TiO_2_+Tm_2_Ti_2_O_7_ structure. Figure 6a shows a TEM image of this sample, with an EELS map of Ti and Tm (grey scale and temperature scale insets). The Ti-related, Ti3 peak (47 eV) is shown as white contrast in the inset of Figure 6a, and the Tm-M_4,5_ signal is presented as yellow contrast in temperature map. These results reveal the formation of Tm clusters in the mapped region. Furthermore, a higher concentration of Tm along the edge of the nanoparticle is revealed in the inset of Figure 6a, indicating an inhomogeneous distribution of Tm in the particle. To verify this result, an EELS linescan of the Tm-M_4,5_ signal (see Figure 6b) was carried out along the nanoparticle (the white arrow in Figure 6a indicates the scan direction). The result of the Tm EELS linescan is presented in Figure 6b, where the blue unfilled dots are used to evaluate the Tm-M_4,5_ intensity along the linescan. The profile shows a strong variation in the Tm EELS intensity, revealing a non-uniform distribution of Tm, and the formation of clusters with a higher concentration of Tm along one edge of the nanoparticle. Moreover, the specific thermal treatment implies the formation of a rutile structure (as can be appreciated in the low-loss spectra of Figure 6b). However, the crystalline structure is not constant along the particle. Figure 6b shows an EELS spectrum acquired in positions (a) and (b) that corresponds to the core and edge of the nanoparticle. It can be noted that the Ti-related peaks (labeled as Ti1–3 in Figure 6b) vary in intensity and position: a low-loss shape for (b) corresponds to a rutile spectra, while low-loss signal for (a) is different, and possibly indicates a mixture of structures along the nanoparticle. This was similar to the results reported for the XRD and Raman results, that is, the sample is composed of rutile, pyrochlore and anatase phases.

**Figure 7:**
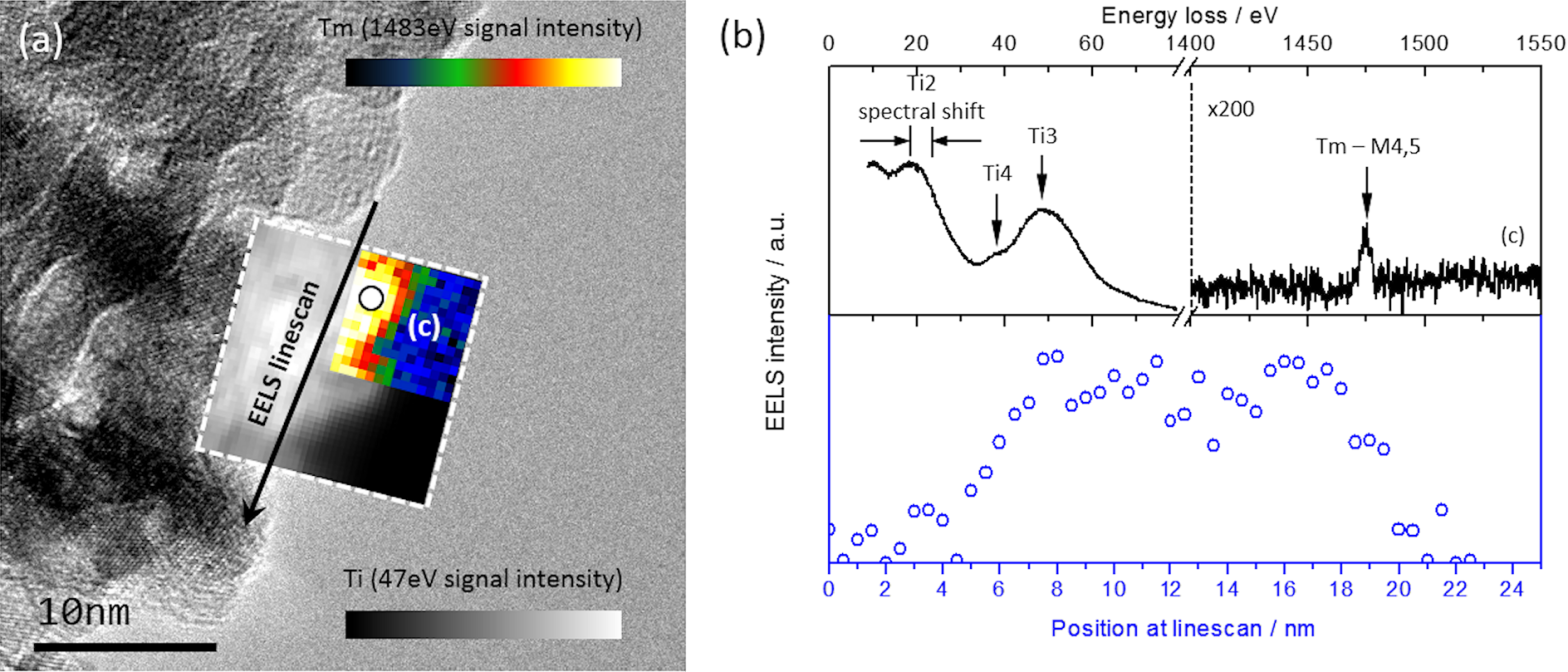
(a) TEM image of the 5.8 atom % Tm-doped sample annealed at 973 K and EELS maps obtained for the Ti3 peak (grey scale map) and Tm-M_4,5_ signal (temperature map). (b) EELS spectra obtained at position (c) of Figure 7a and the Tm-M_4,5_ signal intensity profile along the corresponding linescan of Figure 7a.

In contrast with the results already shown in Figure 6, Figure 7 shows a Tm-doped nanoparticle from a 5.8 atom % Tm-doped sample annealed at 973 K, whose EELS Tm map indicates a homogeneous distribution inside the Tm particle. To verify this result, an EELS linescan of the Tm-M_4,5_ signal was carried out along the edge of the nanoparticle (black arrow in Figure 7a indicates scan direction). The result of the Tm profile is presented in Figure 7b with blue unfilled dots; the profile shows a uniform Tm distribution along the 25 nm linescan, with no evidences of cluster formation. On the other hand, the low-loss spectra presented in Figure 7b shows Ti spectral shifts with respect to the peaks presented in Figure 6a, and a new peak (Ti4) characteristic of the anatase structure is revealed. The general shape of the low-loss spectra is closer to that of the anatase phase, and therefore, the sample shows a more specific presence of anatase structure (in contrast to that of the 2.0 atom % Tm-doped sample annealed at 1173 K, Figure 6).

### Photocatalytic activity

**Figure 8:**
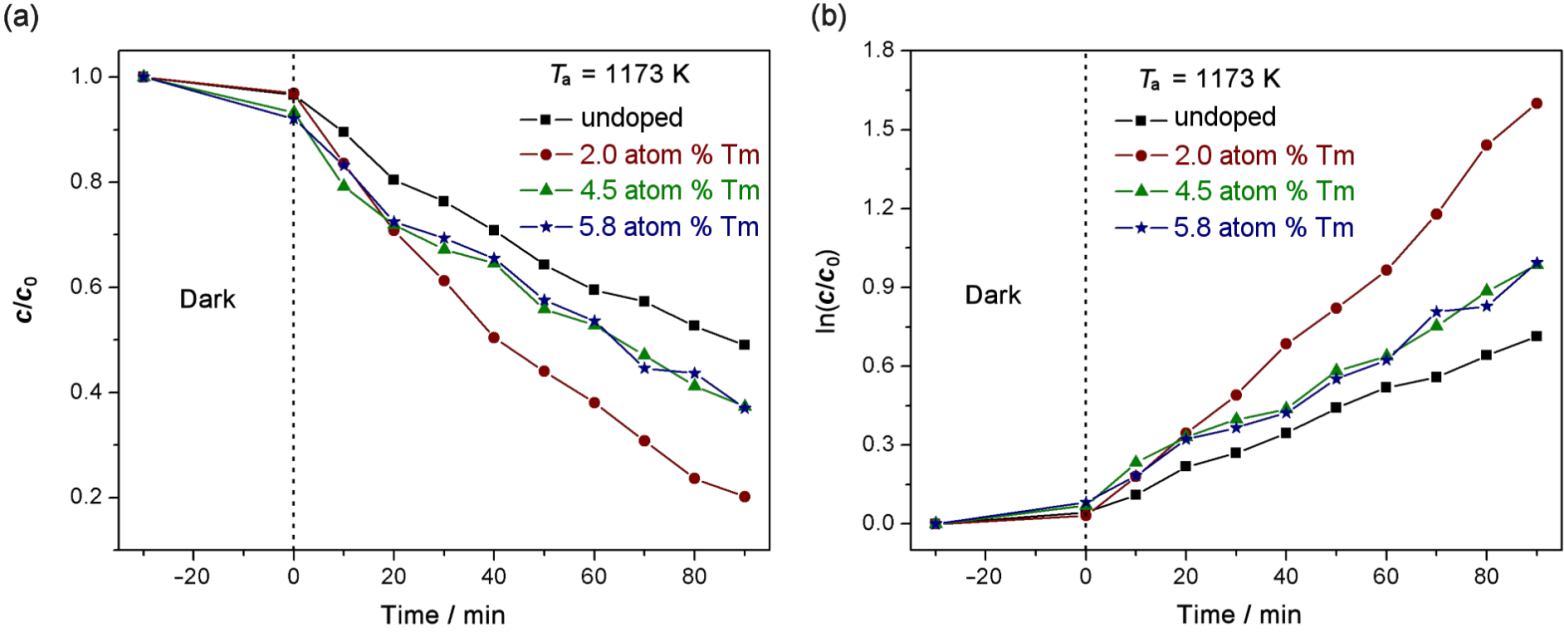
Photodegradation of MB under UV irradiation using the samples annealed at 1173 K as catalyst.

Photocatalytic activity tests were performed to study the effect of the pyrochlore phase on the photocatalytic activity of rutile TiO_2_. A reference experiment without a catalyst was performed and no significant degradation of the MB was found. Figure 8 shows the time evolution of the degradation of MB using the samples annealed at 1173 K as a photocatalyst. It shows that the doped samples have greater photocatalytic activity than pure TiO_2_. At this annealing temperature, the doping and synthesis methodology used produces a pyrochlore phase (Tm_2_Ti_2_O_7_) together with a rutile phase TiO_2_ for a 5.8 atom % Tm-doped sample and a mixture of pyrochlore, rutile and anatase phases for 2.0 and 4.5 atom % Tm-doped samples (where rutile is the predominant phase, at 87% and 94% respectively, Table 2). This implies that the photocatalytic properties of the semiconductor can be improved by adding a pyrochlore phase to the rutile phase as is shown for the 5.8 atom % Tm-doped sample results. The 2.0 atom % Tm-doped TiO_2_ sample presented the best photocatalytic activity. Furthermore, in accordance with the results obtained (Figure 8b), the photocatalytic degradation of MB, using our samples synthesized as a catalyst, is a pseudo-first-order reaction and its kinetics can be described by ln(*c*_0_/*c*) = −*kKt* = *k*_app_*t*, where *c*_0_ is the initial concentration of the MB, *c* is the concentration of the MB with irradiation time *t*, *k* is the reaction rate constant, *K* is the adsorption constant of the reactant, and *k*_app_ is the apparent rate constant [2]. Moreover, the half-life time is calculated for the pseudo-first-order reaction as *t*_1/2_ = 0.693/*k*_app_. The values of *k*_app_ and *t*_1/2_ for the photodegradation tests observed in Figure 8 are shown in Table 3. From these values, it is shown that the tests performed using the 2.0 atom % Tm-doped TiO_2_ samples as a catalyst were 2.35 times faster than the pure TiO_2_ sample with respect to the photodegradation of the MB.

**Table 3:** Values for *k*_app_ and *t*_1/2_ obtained for the photocatalytic activity tests performed.

Catalyst annealed at 1173 K	*k*_app_/min^−1^	*t*_1/2_/min

Pure TiO_2_	0.0074	93.6
2.0 atom % Tm-doped TiO_2_	0.0174	39.8
4.5 atom % Tm-doped TiO_2_	0.0096	72.2
5.8 atom % Tm-doped TiO_2_	0.0097	71.4

Photocatalytic activity depends on many properties of the catalyst, including the band gap energy, the specific surface area, the surface structure, extent of crystallinity, and the structure of the material. The differences in the band gap values for the four catalysts used were very small. Additionally, the difference in the specific surface area between the pure TiO_2_ sample and the 2.0 atom % Tm-doped TiO_2_ sample was minimal (Table 2 and Figure 4), and these factors alone cannot explain the difference in the photocatalytic activity between the samples synthesized. The degree of crystallinity is similar for the two samples, as evidenced by the XRD patterns (Figure 1). However, the structure of the material is different, and this could be a reason for the difference in photocatalytic activity. The 2.0 atom % Tm-doped TiO_2_ semiconductor is composed of three phases, rutile, anatase and pyrochlore. It is known that photocatalytic activity is controlled by the charge separation efficiency. The process of catalytic photodegradation of organic compounds can be described as follows: a continuous irradiation (with an energy higher than band gap energy) of an aqueous semiconductor dispersion excites an electron from the valence band to the conduction band, creating an electron–hole pair [10]. The photogenerated holes can oxidize H_2_O or OH^−^ to give OH^•^ [13], or to oxidize the organic compound directly. On the other hand, the photogenerated electrons could reduce O_2_ to give O_2_^•^. One of the main objectives in improving photocatalytic activity is the suppression of the recombination of the electron–hole pairs, because the recombination is usually much easier than the subsequent steps required for organic degradation [10]. In our case, the heterojunction that exists at the interface of the mixed phases in the doped samples promotes charge separation, that is, the separation of electron–hole pairs. This has been reported previously for NaTaO_3_/Na_2_Ta_2_O_6_ phases [43]. An efficient charge separation implies low recombination, and this has been observed from the Raman results for the pyrochlore-based samples. Moreover, the higher photocatalytic activity in the ordered pyrochlore may be due to the increased mobility of electrons through Ti–O–Ti bonds in the Tm_2_Ti_2_O_7_ lattice. Previous luminescence studies have shown that the closer the M–O–M bond angle is to 180°, the more delocalized the excited state [12,44], and the easier it is for charge carriers to move within the matrix. The mobility of electrons and photogenerated holes affects the photocatalytic activity because the likelihood of electrons and holes reaching reactive sites in the catalyst surface is increased by high diffusivity [12]. In our case, the bond angle of Ti–O–Ti in Tm_2_Ti_2_O_7_ is close to the ideal 180° typical for pyrochlore-type structures. In this case, the movement of photogenerated electrons is improved and the recombination of electron–holes pairs is decreased, thus enhancing the photocatalytic activity.

Thus, the photocatalytic activity was improved when catalysts of mixed phase are used, which occurred to a greater extent for the 2.0 atom % Tm-doped TiO_2_ sample. This is because the presence of the interface between rutile, anatase and pyrochlore improves the separation of the electron–hole pairs generated. The high degree of mobility of the electrons in the pyrochlore structure results in a reduction of recombination process. These two factors improve the photocatalytic activity of this semiconductor when compared with pure rutile TiO_2_. For the 5.8 atom % Tm-doped sample, only rutile and pyrochlore phases are obtained, and the photocatalytic activity is improved with respect to the pure rutile TiO_2_. This is evidence of the positive effect of the pyrochlore phase on the photocatalytic activity of the semiconductors synthesized.

Therefore, from the results reported, a mechanism for the degradation of the MB is proposed as follows. When TiO_2_ is irradiated under UV excitation, the photoinduced electrons and holes produce a reaction with attached molecules on the material surface and create reactive radicals. The electrons in the conduction band react with O_2_ to form superoxide anion radicals (O_2_^−•^) and the holes in the valence band react with water to form hydroxyl radicals (^•^OH). The activated radicals play a role in degrading pollutants [45]. It is generally suggested that the rate-limiting steps of the photocatalytic reaction of semiconductors are electron–hole recombination and electron transfer from the semiconductor surface to the adsorbed oxygen molecules [45–48]. In our case, the pyrochlore phase can trap electrons from the conduction band of the semiconductor to reduce the electron–hole recombination, and to improve the charge separation efficiency. The decrease of the electron–hole recombination increases the photocatalytic activity of the catalysts.

## Conclusion

This study presents the synthesis of Tm-doped TiO_2_. The resulting phases found in the samples differed in accordance with the annealing temperature. For the samples annealed at 773 and 973 K, an anatase phase TiO_2_ was obtained, predominantly doped with Tm^3+^ up to 5.8 atom %. In the samples annealed at 1173 K, a mix of TiO_2_, predominantly a rutile phase, and a pyrochlore phase corresponding to the mixed oxide Tm_2_Ti_2_O_7_ was obtained. In both cases, the presence of Tm^3+^ was confirmed by XPS and UV–vis spectroscopy. The UV–vis spectra showed new absorption bands in the visible part of the electromagnetic spectrum, which are assigned to the presence of Tm^3+^. Finally, photodegradation tests of methylene blue were performed with the samples annealed at 1173 K, which were composed of rutile and pyrochlore phases. The reaction rate increased by a factor of 2.35 in the sample with 2.0 atom % of Tm. Thus, the photocatalytic activity of the rutile phase TiO_2_ is greatly improved with the presence of a pyrochlore phase.
